# Turtle-Associated Salmonellosis, United States, 2006–2014

**DOI:** 10.3201/eid2207.150685

**Published:** 2016-07

**Authors:** Stacey Bosch, Robert V. Tauxe, Casey Barton Behravesh

**Affiliations:** Centers for Disease Control and Prevention, Atlanta, Georgia, USA

**Keywords:** Salmonella infections, turtles, zoonoses, outbreaks, bacteria, salmonellosis, Salmonella spp., United States, human salmonellosis

## Abstract

During 2006–2014, a total of 15 multistate outbreaks of turtle-associated salmonellosis in humans were reported in the United States. Exposure to small pet turtles has long been recognized as a source of human salmonellosis. The risk to public health has persisted and may be increasing. Turtles are a popular reptilian pet among children, and numerous risky behaviors for the zoonotic transmission of *Salmonella* bacteria to children have been reported in recent outbreaks. Despite a long-standing federal ban against the sale and distribution of turtles <4 in (<10.16 cm) long, these small reptiles can be readily acquired through multiple venues and continue to be the main source of turtle-associated salmonellosis in children. Enhanced efforts are needed to minimize the disease risk associated with small turtle exposure. Prevention will require novel partnerships and a comprehensive One Health approach involving human, animal, and environmental health.

## Abstract 2

Enhanced efforts are needed to minimize the risk of human salmonellosis acquired from small pet turtles.

*Salmonella* spp. cause an estimated 1.2 million human illnesses, 23,000 hospitalizations, and 450 deaths each year in the United States (*1*). Infections are usually acquired through direct or indirect exposure to contaminated food or animals that carry *Salmonella*, including turtles and other reptiles (*1*,*2*). Most of these infections are foodborne, although an estimated 11% of *Salmonella enterica* infections were recently attributed to animal exposure (*2*). Exposure to small turtles (Figure) has been recognized as a source of human salmonellosis in the United States since the 1960s, when small baby turtles first became a popular pet (*3*). By the early 1970s, ≈15 million turtle hatchlings were sold annually in the United States, 4% of all US households owned at least 1 pet turtle at a given time, and 14% of human salmonellosis cases were attributed to exposure to small pet turtles (*4*). In 1975, to prevent turtle-associated salmonellosis among children, the US Food and Drug Administration (FDA) enacted a ban prohibiting the intra- and interstate sale and distribution of turtles with a shell length of <4 in (<10.16 cm) within the United States; after this ban, the small turtle industry turned to the export trade (*5*–*7*). The federal ban was effective, preventing an estimated 100,000 cases of turtle-associated salmonellosis in children each year after its enactment (*8*). By the late 1990s, only 6% of sporadic *Salmonella* spp. infections in the United States were attributed to reptile and amphibian contact (*9*). However, the regulation allows for small turtles to be distributed for bona fide scientific and exhibition purposes and for educational purposes other than use as pets.

**Figure:**
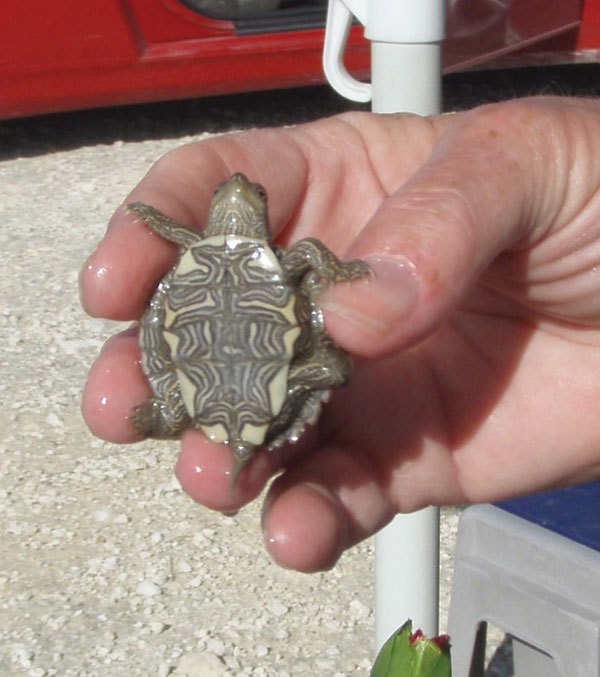
Small turtle with a shell length of <4 in (<10.16 cm). Photo credit: Casey Barton Behravesh.

The risk of acquiring a *Salmonella* infection from turtles has persisted and may be increasing, as suggested by a recent surge in the number of national salmonellosis outbreaks. The increased number of cases indicates the need for renewed attention to this long-standing public health issue, using a One Health approach involving human, animal, and environmental health.

Healthy turtles carry *Salmonella* spp. as part of their normal intestinal flora and intermittently shed the bacteria in their droppings. Humans become infected through direct contact with a turtle or by contact with its habitat, including contaminated tank water (*10*,*11*). Human salmonellosis typically causes acute gastroenteritis; however, severe invasive illness (e.g., sepsis, septic arthritis, meningitis) and death may occur, especially in persons at high risk (e.g. children <5 years of age, seniors, pregnant women, and immunocompromised persons). Turtle-associated salmonellosis disproportionately affects persons at high risk for severe illness, particularly infants and young children (*3*,*4*,*12*–*16*).

## Increase in Multistate Outbreaks of Turtle-Associated Salmonellosis

Turtle-associated salmonellosis outbreaks were defined as >2 culture-confirmed human *S. enterica* infections with a combination of epidemiologic, laboratory, or trace-back evidence linking the illnesses to turtles. During 2006–2014, a total of 15 multistate turtle-associated salmonellosis outbreaks were reported to and investigated by the Centers for Disease Control and Prevention (CDC; Atlanta, GA, USA); this number represents an average of 2 cases per year. The outbreaks accounted for 921 illnesses, 156 hospitalizations, and the death of a 3.5-week-old infant (Table) (*6*,*12*–*15*,*17*). Outbreaks ranged in size from 4 to 135 (median 44) laboratory-confirmed cases. In all 15 outbreaks, the median age of ill persons was <10 years, indicating that children are still the most affected by turtle-associated salmonellosis.

**Table:** Characteristics of 15 multistate outbreaks of human *Salmonella enterica* infections linked to turtle exposure, United States, 2006–2014*

Outbreak no., year†	Duration, mo	Serotype(s)	Outbreak strain(s)‡	No. cases	No. states	No. hosp.	No. deaths	Median patient age, y (range)
1, 2006	1	I 4,[5],12:i-	JPXX01.0621, JPXX01.1056	4	2	1	0	10.0 (7–45)
2, 2007	7	Pomona	POMX01.004, POMX01.002	20	11	1	1	3.0 (<1–59)
3, 2007	4	Paratyphi B var. Java	JKXX01.0014, JKXX01.0015, JKXX01.0038	107	34	26	0	7.0 (1–87)
4, 2008	8	Typhimurium	JPXX01.0416, JPXX01.0006	135	25	29	0	7.0 (1–94)
5, 2009	5	Muenchen	JJ6X01.0063	10	8	0	0	10.0 (<1–60)
6, 2011	14	Paratyphi B var. Java	JKXX01.0116	132	18	13	0	6.0 (1–75)
7, 2012	30	Sandiego	JLXX01.0053	124	22	15	0	6.0 (<1–85)
		Newport	JJPX01.1253					
8, 2012	25	Pomona	POMX01.0004	23	14	5	0	5.5 (<1–89)
9, 2012	27	Poona	JLX6X01.0104	58	22	16	0	3.5 (<1–84)
		Sandiego	JLXX01.0002					
10, 2012	8	Sandiego	JLXX01.0051	7	3	1	0	10.0 (<1–65)
11, 2012	32	Pomona	POMX01.0002	120	29	19	0	2.0 (<1–94)
12, 2012	20	Poona	JL6X01.0055	78	13	8	0	3.0 (<1–83)
13, 2012	4	I 4,[5],12:i-	JPXX01.1056	19	5	3	0	2.0 (<1–33)
14, 2012	8	Typhimurium	JPXX01.1048	44	11	11	0	3.0 (<1–70)
15, 2014	7	Poona	JL6X01.0055	40	12	8	0	5.0 (<1–75)

*Hosp., hospitalizations. †Outbreaks are depicted in the year they were reported to the Centers for Disease Control and Prevention. For some, onset of illness may have occurred in preceding years. ‡Defined by pulsed-field gel electrophoresis *Xba*I restriction enzyme pattern.

The 8 multistate outbreaks reported in 2012 alone accounted for 473 reported illnesses; total estimated medical costs were ≈US $2,800,000 (*17*). Among 191 persons for whom information was available, 85 (45%) reported Hispanic ethnicity. Most reported turtles were small: 124 (88%) of 141 ill persons with turtle exposure reported that the implicated turtles had shell lengths of <4 in. Of 35 patients specifically asked their reason for purchasing a small turtle, all reported purchasing the turtle as a pet (*17*).

Patient interviews conducted during these recent outbreak investigations indicated that knowledge of the connection between reptiles and salmonellosis was lower than in previous turtle-associated outbreaks. Only 14 (15%) of 95 patients who reported turtle exposure during the 2012 outbreaks were aware that reptiles could carry *Salmonella* bacteria (*17*). By comparison, 20% of patients in the 2007–2008 *Salmonella* Paratyphi B var. Java outbreak investigation and 27% in the 2008 *Salmonella* Typhimurium outbreak investigation knew of the connection (*13*,*14*). This observation is concerning because numerous risky behaviors were reported in the 2012 outbreaks, including kissing turtles, letting them roam on kitchen countertops and tabletops where food and drink was prepared or consumed, and cleaning turtle habitats in kitchen sinks, all of which can lead to transmission of *Salmonella* bacteria. Frequency of reported turtle contact behavior and knowledge of the connection between reptiles and salmonellosis did not vary by reported ethnicity (*17*).

As part of the 2012 investigations, multiple federal and state public health and regulatory agencies collaborated to trace small turtles, which had been illegally sold in Florida beachside souvenir shops, back to 2 turtle farms in Louisiana. Two different outbreak strains were isolated from 1 farm’s breeding pond: *Salmonella* Pomona (PFGE *Xba*I restriction enzyme pattern POMX01.0004) and Poona (PFGE *Xba*I restriction enzyme pattern JLX6X01.0104) (*16*). Because turtle farms in Louisiana are regulated by the Louisiana Department of Agriculture and Forestry (*18*), cease and desist orders were issued on domestic shipments of turtles from the implicated farms, thereby stopping, at the source, distribution of the turtles causing human illness. The Florida Department of Health and the Florida Wildlife Conservation Commission stopped the sale of small turtles at the souvenir shops, highlighting the effectiveness of state agency actions in these investigations (*17*).

## Trends in Turtle Ownership and Sources of Turtles

The increase in turtle-associated salmonellosis may be related to the growing popularity of turtles as pets in the United States over the past 15 years. The proportion of US households that own pet turtles increased from 0.5% in 1996 to 1.1% in 2011 (*19*). Turtles are the most common reptile species owned as pets in the United States; approximately twice as many households own turtles than own pet snakes (0.5%) or lizards (0.6%) (*19*). No national data indicate what proportion of pet turtles have shell lengths <4 in, although a resurgence in the illegal sale and distribution of small pet turtles was reported by the FDA in 2003–2004 (*20*,*21*).

Small turtles can be purchased from retail pet stores, discount stores, flea markets, swap meets, roadside vendors, street vendors, beachside souvenir shops, and online merchants (*6*,*17*). In addition, small turtles are often available for sale at fairs, outside of sporting events, or at parks. Because small turtles are being sold illegally, it is difficult to quantify how many are purchased as pets in the United States; however, it appears they come primarily from domestic farmers and distributors. According to the US Fish and Wildlife Service, ≈1.6 million turtles of any size were imported into the United States during 2000–2011 (US Fish and Wildlife Service, pers. comm., 2012 May 15); this number represents a small fraction of the ≈151 million turtles exported to other countries during the same period. Furthermore, during 2006–2012, US quarantine stations detained and denied US entry only 7 times to shipments of turtles with shell lengths of <4 in that were imported for commercial purposes. Together, those 7 shipments totaled 66 turtles (CDC, Division of Global Migration and Quarantine, pers. comm., 2013 Mar 23).

## Special Risk of Small Turtles

The regulatory size restriction for turtles (i.e., length <4 in) was designed to protect children without interfering with the desire of turtle fanciers to obtain larger turtles (*6*). Small turtles are inexpensive to purchase and may seem to be a safe and attractive pet for young children. Indeed, they are more likely to be given as pets to children compared with other reptiles, such as snakes and iguanas (*6*,*22*), because small turtles are perceived as harmless, slow-moving pets that are safe for children. Hatchlings are small enough to fit in a young child’s mouth and are also kissed and held in close physical contact by their young owners (*6*,*17*). Small turtles are often housed in a small pool of water in a plastic turtle bowl, which can become heavily contaminated with *Salmonella* spp. (*4*,*11*). Illnesses have also been attributed to swimming with turtles in an unchlorinated pool (*12*). Cleaning turtle habitats in a kitchen sink or bathtub can lead to cross-contamination with *Salmonella* bacteria and indirect transmission to persons who never had direct contact with the small turtle; this scenario is common for infants who become infected (*17*). Even diligent caregivers can have difficulty ensuring that young children wash their hands immediately and properly after handling a pet turtle or its habitat.

## Changes in Turtle-Farming Practices

The US turtle-farming industry has supported research into methods to reduce the carriage rate of *Salmonella* spp. in pet turtles. These efforts have been driven primarily by Louisiana, the only state that currently licenses and regulates its turtle farms (*18*). Farms in Louisiana that sell turtles domestically or internationally must meet certain sanitary conditions, and they are required to treat turtle eggs with a surface disinfectant wash followed by a treatment with a bactericidal solution delivered through the egg pores via a pressure-differential process. Louisiana turtle farms are also required to undergo routine facility and equipment inspections at least once a year. In addition, state inspectors from the Louisiana Department of Agriculture and Forestry collect a 1-time random sample of 60 eggs or hatchling turtles from each lot (≈20,000 eggs or hatchlings) intended for sale. These samples are then tested for *Salmonella* spp*.* at a state-approved laboratory. If a *Salmonella* sp. is identified in the eggs or turtles sampled, that lot is removed from commerce (*18*). This process has prompted some farmers who followed this protocol and passed state inspections to incorrectly claim they are selling certified *Salmonella*-free turtles.

Academic researchers have found bactericidal pressure-differential egg treatment methods reduce but do not eliminate the frequency and quantity of *Salmonella* spp. in turtle hatchlings (*23*–*25*). Little is known about how those efforts affect *Salmonella* spp. carriage rates in turtles at points of sale or in household aquariums. In a recent study, researchers following *Salmonella-*free turtle hatchlings for 1 year in a laboratory setting found they did not shed *Salmonella* bacteria during that time (*26*). That study was conducted using turtle hatchlings acquired directly from a turtle farm and then housed in controlled aquarium environments with ideal husbandry practices (e.g., using an in-tank water circulator and bioscrubber, providing consistent lighting, and feeding a consistent commercial diet). Similar long-term studies using treated egg–hatched turtles that are sold and raised in homes are lacking; in such a study, the turtles would be shipped to retail stores in a box with many other turtles and then held in store tanks before being purchased by consumers who use a variety of housing environments and feeding practices. This information gap is critical because multiple outbreaks of human illness have been attributed to turtles that were claimed to be certified *Salmonella*-free, including the 2012 multistate outbreak of serotypes Poona and Sandiego (Table) (*17*,*27*).

Although the turtle farming industry has changed since the 1960s, in light of recent investigation findings, we caution against the overreliance on egg treatment methods alone to reduce turtle-associated salmonellosis. Advertisement of a *Salmonella*-free turtle could give consumers a false sense of security, making them less likely to wash their hands or think other precautions are necessary after handling a turtle or its habitat. Even if turtles do not carry *Salmonella* spp. at the time of sale, they might not remain *Salmonella*-free throughout their lives (*6*). *Salmonella* bacteria are ubiquitous in the environment and are natural inhabitants of the turtle gastrointestinal tract. Turtles could acquire *Salmonella* spp. through several routes, including from other turtles through cross-contamination during shipping or comingling in holding tanks or through contaminated food. Turtles from multiple sources are often kept in high-density conditions in store tanks, which may not be regularly cleaned or maintained (*28*). No market controls or industrywide guidance promote humane and proper housing of turtles in stores after they leave the farm; this is another area for improvement because rates of *Salmonella* spp. shedding are probably higher among turtles housed in stressful conditions (*24*,*29*).

## Ongoing Federal Ban Enforcement Challenges

Since the federal ban against the sale of small turtles was enacted, turtle producers have sought to repeal the ban, including through proposed federal legislation and lawsuits in federal court (*30*,*31*). Although the federal ban remains in place, its enforcement continues to be challenging. Consumer demand for baby turtles has led to a veritable black market of small turtles. Many of these turtles are purchased with cash from transient, untraceable vendors, such as sellers in flea markets and unmarked vans or roadside vendors. Therefore, any subsequent regulatory action by state or federal agencies would be difficult or impossible to conduct (*6*,*12*–*15*,*17*). Turtles sold via the Internet and shipped through the mail may also be difficult to trace.

Some merchants routinely exploit a regulatory exemption allowing for the purchase of small turtles for “bona fide scientific, exhibition, or educational purposes, other than use as pets.” This is done by asking customers to sign a waiver stating they are purchasing a small turtle for educational purposes only (*21*,*27*,*28*). Some vendors on the Internet provide information on the illegality of selling small turtles as pets and a person’s risk for *Salmonella* infection buried in the fine print of the website’s terms and conditions of use (*6*). Customers are asked to check a box indicating they have read and agree to the terms of use when purchasing turtles online; this effort is dubious because <10% of customers on the Internet read terms of use agreements when purchasing products online (*32*). It seems that few persons in the United States who purchase small turtles over the Internet are likely to know that they are purchasing an illegally sold product that could make them sick. A bona fide market for turtles purchased for scientific and educational purposes may exist, but in outbreak after outbreak, ill persons reported acquiring their small turtles specifically as pets, an act prohibited under the federal ban (*6*,*12*–*15*,*17*,*27*).

Lack of regulatory authority at the state or local level creates another hurdle in stopping the sale of small turtles. A review of state laws in March 2014 identified 10 states with regulations prohibiting or restricting the sale of turtles with shells <4 in long that enable those states to pursue enforcement activities against the sale of small turtles in their jurisdictions (*33*). States that have not enacted such laws are reliant upon the FDA to enforce regulations, but federal resource limitations mean the FDA must prioritize which turtle suppliers to investigate and prosecute. States may wish to develop their own regulations limiting or banning the sale of small turtles, including requirements that merchants display signage on the human health risks of reptile ownership and barring all turtles from nursing homes and schools and daycare facilities serving young children (*6*,*34*,*35*). Some states have enacted new laws regulating small turtles (*6*,*33*,*34*), but others have encountered challenges (J. Scheftel, pers. comm., 2014 Mar 31), indicating a need to identify other ways to empower state and local jurisdictions to prevent illegal turtle sales.

In states without laws prohibiting the sale of small turtles, investigators have asked retail merchants to voluntarily stop illegal turtle sales in response to outbreaks of human salmonellosis (*17*,*27*). Public health investigators have partnered with other state agencies (e.g., Departments of Agriculture or Fish and Wildlife) with enforcement authorities over the sale of animals (e.g., prohibitions against the sale of endangered or invasive species) (*17*,*27*). In addition, the pet industry has a role to play in confronting this public health issue.

## Retail Pet Industry

Small turtles implicated in outbreaks were often purchased from small, independently owned retail pet stores whose proprietors often claimed to have no knowledge of the federal ban (*12*,*13*,*17*,*27*). By contrast, national pet store chains typically do not sell turtles with shell lengths <4 in because doing so is illegal and because hatchlings have poor survival rates in store tanks and tanks in customers’ homes (T. Edling, pers. comm., 2014 Mar 20). An opportunity exists to educate small pet store owners and engage their help in FDA ban compliance. Public health agencies can send letters to licensed pet store owners, informing them of turtle-associated *Salmonella* infections reported in their area and of the ongoing federal ban against the sale and distribution of turtles with shell lengths <4 in. In addition, public health agencies can inform pet store owners of any applicable state laws or local laws and ask them to prominently post education materials on the risk of *Salmonella* infection from reptiles.

## Conclusions and Recommendations

The long-standing public health issue of turtle-associated salmonellosis is reemerging in the United States, where multistate outbreaks have increased since 2006. These illnesses have most often occurred after exposure to small pet turtles with shell lengths <4 in, the sale and distribution of which is illegal in the United States. Further efforts to prevent salmonellosis from pet turtles will take an integrated One Health approach involving human, animal, and environmental health officials as well as the turtle industry and the retail pet industry.

Public health partners can help spread awareness, in English and Spanish, of the risk of turtles as a source of salmonellosis in humans and the particular hazard small turtles pose for young children. Pediatricians and family practice physicians are in a unique position to educate families about the risk for turtle-associated salmonellosis during wellness examinations for young children. Veterinarians can reinforce these messages by recommending reptiles as pets only for households with children >5 years of age and by providing detailed instruction to clients on proper reptile care and practices to prevent zoonoses. Healthcare providers for humans and animals can make educational literature available in waiting rooms and provide information on websites and in newsletters (*36*,*37*). Suitable educational materials are available in multiple formats and languages on the CDC Zoonotic Diseases (Diseases from Animals) website (http://www.cdc.gov/zoonotic/gi). If pediatricians have a young patient with salmonellosis, they should consider reptiles in the differential of exposures and inform the local health department if small turtles appear to be involved.

In accordance with federal law, turtle farmers, pet store owners, souvenir shop owners, and others who sell turtles should not sell or distribute those with shells <4 in long. Collaboration between human and animal health officials at state- and federal-level public health and regulatory agencies is often necessary to identify and stop merchants and suppliers who illegally sell small turtles. When state and local authorities are able to investigate suppliers, any regulatory action can be facilitated by the collection of water and environmental samples for culture as well as affidavits, bills of lading and invoices, photos to verify turtle size and breed (e.g., turtle pictured next to a ruler or quarter), and receipts that show purchase of small turtles.

Merchants who legally sell or display turtles (i.e., turtles with shell lengths >4 in and that are not endangered or otherwise prohibited from sale) can serve as positive role models in the effort to reduce the incidence of turtle-associated salmonellosis. Merchants should use good turtle husbandry practices to reduce in-store stress to minimize *Salmonella* spp. shedding and spread among turtles in the store. These practices could include maintaining a low turtle density in tanks, using a reputable turtle supplier, avoiding the mixing of turtles from different sources, using a water recirculator and filter, and feeding with a *Salmonella*-free food (T. Edling, pers. comm., 2014 Apr 15). In addition, merchants can prominently display information in stores and online about the risk of acquiring salmonellosis from turtles (as well as other reptiles and amphibians) and their tanks or aquariums and instructions for proper cleaning of the turtle habitat. Pet store staff educated about the risk of salmonellosis can direct customers to a more appropriate pet if persons at high risk for severe illness are in the household. This information should be provided to customers well before the point of purchase, not at the cash register or buried in terms of use agreements. CDC and other public health officials are partnering with reptile hobbyist and tradeshow groups and representatives from the pet industry to engage their participation in developing an integrated approach for keeping illegal turtles out of the marketplace.

Turtle-associated salmonellosis remains a preventable and costly public health problem almost 50 years after it was first recognized in the United States. Enhanced efforts to minimize the risk associated with small turtles are needed, including novel One Health partnerships and approaches for prevention.
